# Supplementary material for: Metaxins are core components of mitochondrial transport adaptor complexes
Source: Nat Commun. 2021 Jan 4;12:83. doi: 10.1038/s41467-020-20346-2 (PMC7782850; doi:10.1038/s41467-020-20346-2)
Supplement: Supplementary file 1 — Supplementary Information [file 41467_2020_20346_MOESM1_ESM.pdf]

Supplementary Information

Metaxins are core components of mitochondrial transport adaptor complexes

Yinsuo Zhao[1,2], Eli Song[1], Wenjuan Wang[1], Chung-Han Hsieh[3], Xinnan Wang[3], Wei Feng[1,2,5], Xiangming Wang[1,5], and Kang Shen[4,5]

This file contains:

- Supplementary Fig. 1
- Supplementary Fig. 2
- Supplementary Fig. 3
- Supplementary Fig. 4
- Supplementary Fig. 5
- Supplementary Fig. 6
- Supplementary table 1
- Supplementary table 2
- Supplementary table 3
- Supplementary table 4

Supplementary Fig. 1

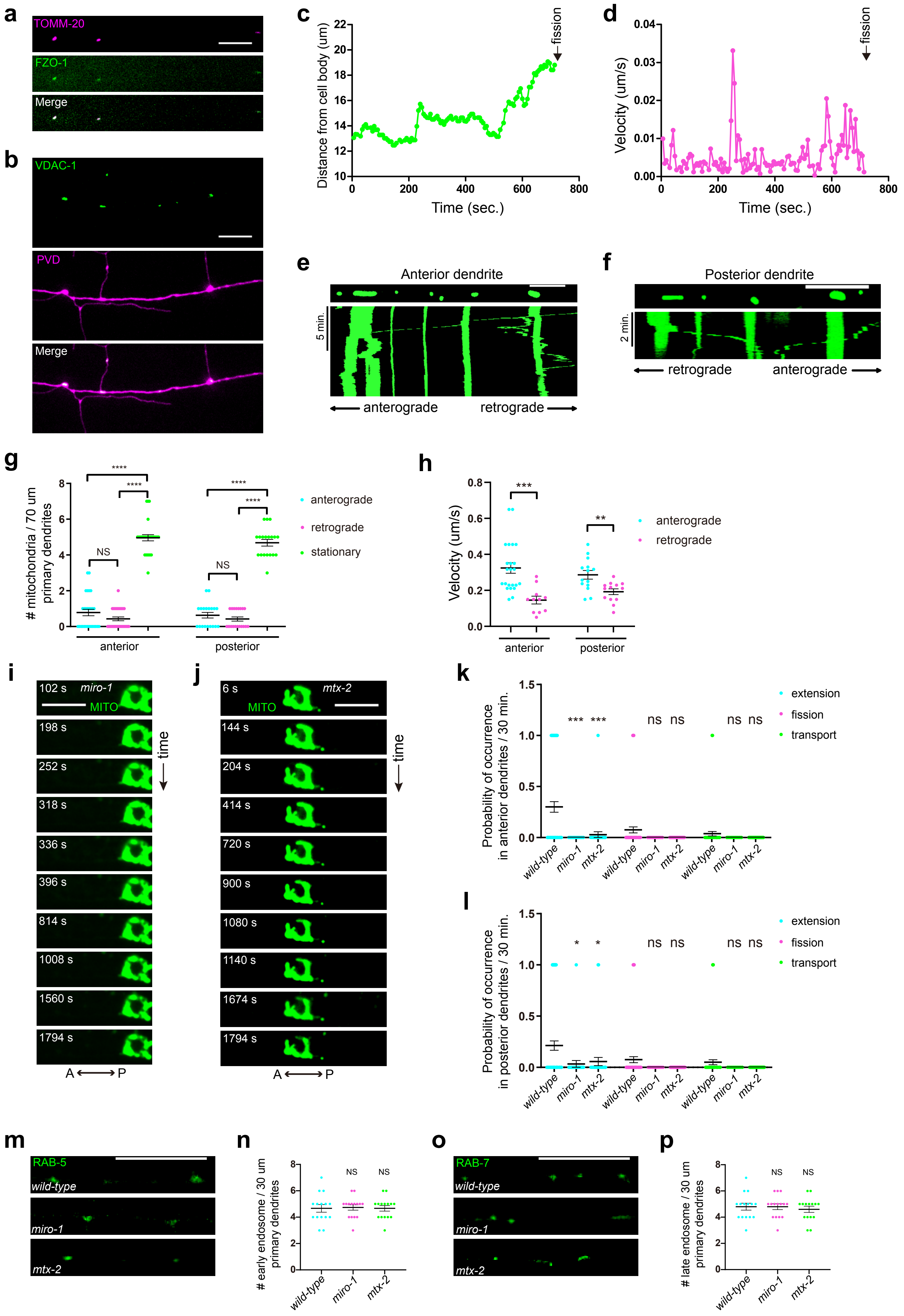

Supplementary Fig. 1: Time-lapse imaging of mitochondrial dynamics in *wild-type*, *miro-1*, and *mtx-2* in PVD dendrite, related to Fig. 1.

a, PVD>FZO-1::GFP transgene co-localizes with PVD>TOMM-20(1-54AA):: mCherry. Scale bar: 10 μm. **b,** VDAC-1::GFP expression pattern in PVD neuron. Scale bar: 10 μm. **c,** Representative mitochondria dynamics showing distance from cell body varies with time. **d,** Representative mitochondria dynamics showing velocity varies with time. **e, f,** Representative kymograph showing anterograde and retrograde mitochondria movement in anterior (**e**) and posterior (**f**) dendrites of PVD. Scale bar: 10 μm. Anterior is to the left. **g,** Quantification of mitochondria dynamics in anterior and posterior dendrites of PVD. Data are shown as mean±SEM. One way ANOVA with Tukey's multiple comparisons test (95% CI). $p>0.05$, not significant, ****$p<0.0001$. (exact p values and sample size: anterior, anterograde vs. retrograde, $p=0.2517$; anterograde vs. stationary, $p<0.0001$; retrograde vs. stationary, $p<0.0001$. anterograde, $n=28$; retrograde, $n=28$; stationary, $n=28$ movies. posterior, anterograde vs. retrograde, $p=0.6109$; anterograde vs. stationary, $p<0.0001$; retrograde vs. stationary, $p<0.0001$. anterograde, $n=19$; retrograde, $n=19$; stationary, $n=19$ movies). **h,** Quantification of mitochondrial velocity of anterograde and retrograde in PVD dendrites. Data are shown as mean±SEM. Student's *t* test (95% CI). ***$p<0.001$, **$p<0.01$. (exact p values and sample size: anterior, anterograde vs. retrograde, $p=0.0004$. anterograde, $n=23$; retrograde, $n=11$ mitochondria. posterior, anterograde vs. retrograde, $p=0.0036$. anterograde, $n=14$; retrograde, $n=13$ mitochondria). **i, j,** Representative time lapse imaging of mitochondria dynamics in *miro-1* (**i**) and *mtx-2* (**j**) mutants in dendrites of PVD. Scale bar: 10 μm. Anterior is to the left. **k, l,** Quantification of mitochondrial dynamics in anterior (**k**) and posterior (**l**) dendrites of PVD in wild type and mutants. Fisher's exact test was used. $p>0.05$, not significant, ***$p<0.001$, *$p<0.05$. $n>30$ 30-minute movies for each genotype. **m, o,** Representative confocal images showing early endosome (GFP::RAB-5) and late endosome (GFP::RAB-7) distribution in PVD neuron in wild type, *miro-1*, and *mtx-2* mutants. Green: PVD>GFP::RAB-5 (**m**), PVD>GFP::RAB-7 (**o**). Scale bar: 10 μm. **n, p,** Quantification of early endosome (**m**) and late endosome (**o**) in PVD neuron in wild type, *miro-1*, and *mtx-2* mutants. Data are shown as mean±SEM. One way ANOVA with Tukey's multiple comparisons test (95% CI). $p>0.05$, not significant. (exact p values and sample size: **n,** *wild-type* vs. *miro-1*, $p=0.9786$; *wild-type* vs. *mtx-2*, $p>0.9999$. $n=15$ animals for each genotype. **p,** *wild-type* vs. *miro-1*, $p>0.9999$; *wild-type* vs. *mtx-2*, $p=0.8271$. $n=15$ animals for each genotype). Source data are provided as a Source Data file.

Supplementary Fig. 2

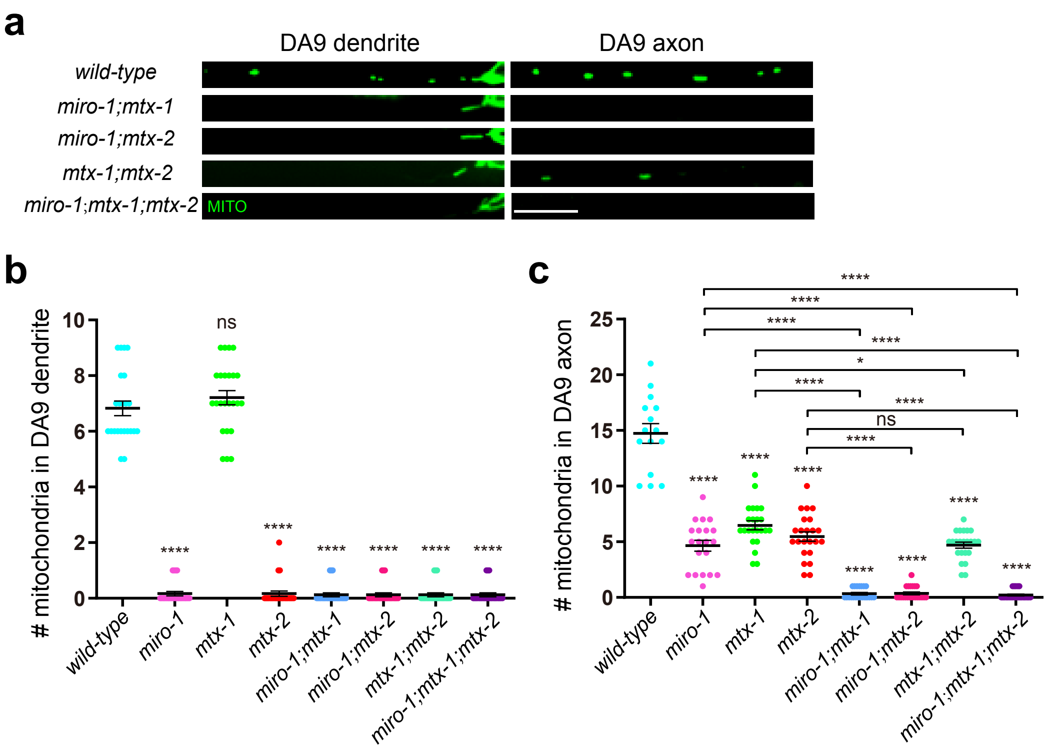

Supplementary Fig. 2: Genetic interaction of metaxins with MIRO-1 in DA9 neuron, related to Fig. 2, 3

a, Representative confocal images showing mitochondrial distribution of DA9 neuron in wild type, *miro-1 mtx-1, miro-1 mtx-2, mtx-1 mtx-2,* and *miro-1 mtx-1 mtx-2* mutants. Green: DA9>TOMM-20 (1-54AA)::GFP. Scale bar: 10 μm. **b, c,** Quantification of mitochondria number in dendrite and axon of DA9 in wild type, *miro-1, mtx-1, mtx-2, miro-1 mtx-1, miro-1 mtx-2, mtx-1 mtx-2,* and *miro-1 mtx-1 mtx-2* mutants. Data are shown as mean±SEM. One way ANOVA with Tukey's multiple comparisons test (95% CI). $p>0.05$, not significant, ****$p<0.0001$, *$p<0.05$. (exact p values and sample size: **b,** *wild-type* vs. *miro-1*, $p<0.0001$; *wild-type* vs. *mtx-1*, $p=0.5777$; *wild-type* vs. *mtx-2*, $p<0.0001$; *wild-type* vs. *miro-1 mtx-1*, $p<0.0001$; *wild-type* vs. *miro-1 mtx-2*, $p<0.0001$; *wild-type* vs. *mtx-1 mtx-2*, $p<0.0001$; *wild-type* vs. *miro-1 mtx-1 mtx-2*, $p<0.0001$. *wild-type*, $n=23$; *miro-1*, $n=24$; *mtx-1*, $n=24$; *mtx-2*, $n=24$; *miro-1 mtx-1*, $n=24$; *miro-1 mtx-2*, $n=24$; *mtx-1 mtx-2*, $n=24$; *miro-1 mtx-1 mtx-2*, $n=24$ animals. **c,** *wild-type* vs. *miro-1*, $p<0.0001$; *wild-type* vs. *mtx-1*, $p<0.0001$; *wild-type* vs. *mtx-2*, $p<0.0001$; *wild-type* vs. *miro-1 mtx-1*, $p<0.0001$; *wild-type* vs. *miro-1 mtx-2*, $p<0.0001$; *wild-type* vs. *mtx-1 mtx-2*, $p<0.0001$; *wild-type* vs. *miro-1 mtx-1 mtx-2*, $p<0.0001$; *miro-1* vs. *miro-1 mtx-1*, $p<0.0001$; *miro-1* vs. *miro-1 mtx-2*, $p<0.0001$; *miro-1* vs. *miro-1 mtx-1 mtx-2*, $p<0.0001$; *mtx-1* vs. *miro-1 mtx-1*, $p<0.0001$; *mtx-1* vs. *mtx-1 mtx-2*, $p=0.0124$; *mtx-1* vs. *miro-1 mtx-1 mtx-2*, $p<0.0001$; *mtx-2* vs. *miro-1 mtx-2*, $p<0.0001$; *mtx-2* vs. *mtx-1 mtx-2*, $p=0.7794$; *mtx-2* vs. *miro-1 mtx-1 mtx-2*, $p<0.0001$. *wild-type*, $n=15$; *miro-1*, $n=20$; *mtx-1*, $n=23$; *mtx-2*, $n=23$; *miro-1 mtx-1*, $n=21$; *miro-1 mtx-2*, $n=22$; *mtx-1 mtx-2*, $n=23$; *miro-1 mtx-1 mtx-2*, $n=23$ animals). Source data are provided as a Source Data file.

Supplementary Fig. 3

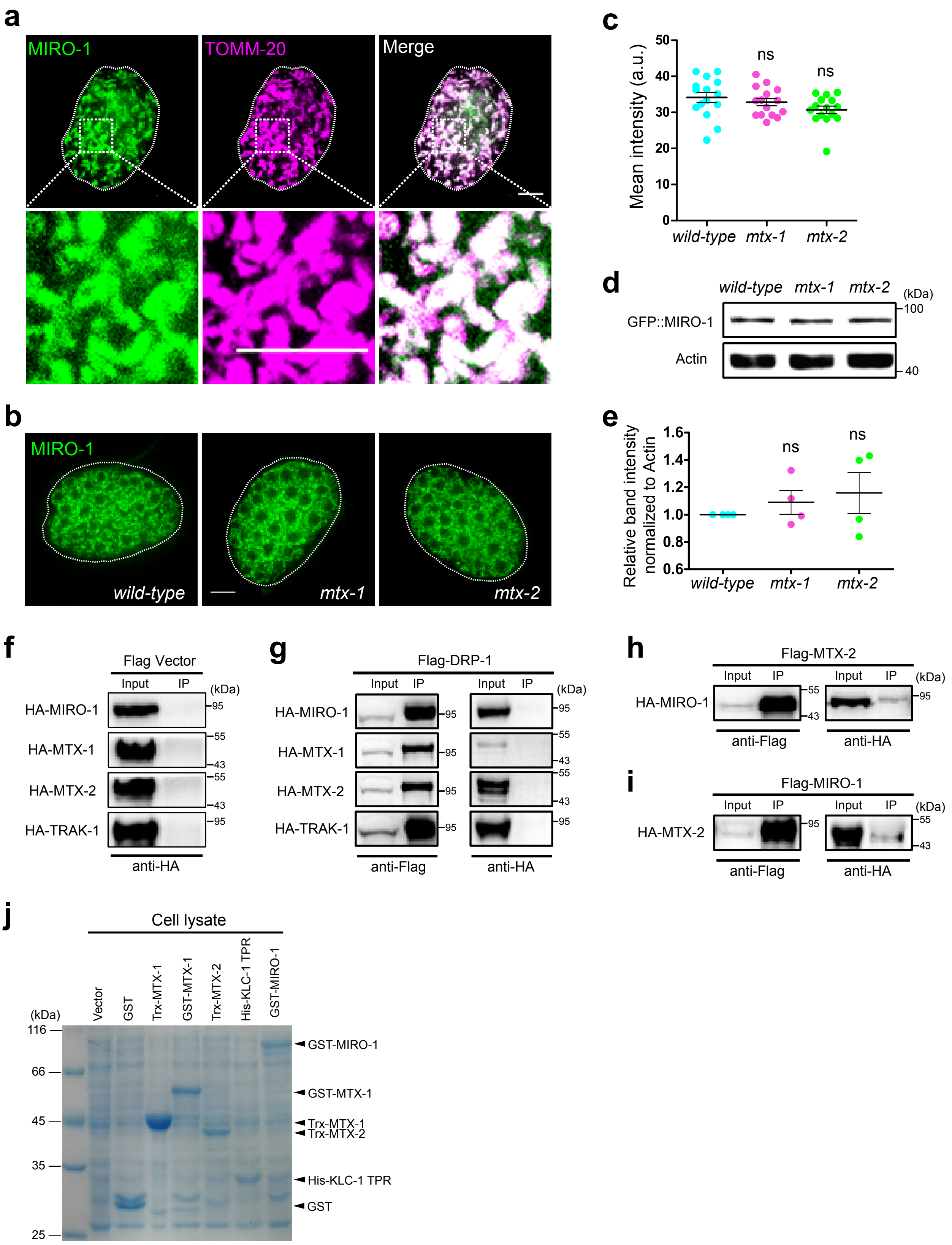

Supplementary Fig. 3: The relationship between metaxins and MIRO-1, related to Fig. 2.

a, Representative confocal images showing MIRO-1 expression pattern (native GFP knock-in), and mitochondrial distribution visualized by co-injected TOMM-20::mCherry. Scale bar: 10 µm. **b,** Representative confocal images showing MIRO-1 expression pattern in wild type, *mtx-2,* and *mtx-1* mutants. Scale bar: 10 µm. **c,** Quantification of GFP::MIRO-1 mean fluorescent intensity in wild type, *mtx-2,* and *mtx-1* mutants. Data are shown as mean±SEM. One way ANOVA with Tukey's multiple comparisons test (95% CI). $p>0.05$, not significant. (exact p values and sample size: *wild type* vs. *mtx-1*, $p=0.7144$; *wild type* vs. *mtx*-2, $p=0.1141$. $n=15$ embryos for each genotype). **d,** Western blot against GFP in wild-type, *mtx-1*, and *mtx-2* mutants with GFP inserted into the N-terminus of the endogenous *miro-1* gene. **e,** Quantification of relative band intensity normalized to actin. Data are shown as mean±SEM. One way ANOVA with Tukey's multiple comparisons test (95% CI). $p>0.05$, not significant. (exact p values and sample size: *wild type* vs. *mtx-1*, $p=0.8041$; *wild type* vs. *mtx*-2, $p=0.5267$. $n=4$ independent experiments). **f, g,** HA-MIRO-1, HA-MTX-1, HA-MTX-2, or HA-TRAK-1 were cotransfected with FLAG vector (**f**), FLAG-DRP-1 (**g**), into HEK293T cells, respectively, and then the cell lysates were immunoprecipitated with ANTI-FLAG M2 affinity gel, followed by western blot analysis with Flag and HA antibodies. $n=3$ independent experiments. **h,** HA-MIRO-1 were cotransfected with FLAG-MTX-2 in HEK293T cells, respectively, and then the cell lysates were immunoprecipitated with ANTI-FLAG M2 affinity gel, followed by western blot analysis with Flag and HA antibodies. $n=3$ independent experiments. **i,** HA-MTX-2 were cotransfected with FLAG-MIRO-1 in HEK293T cells, respectively, and then the cell lysates were immunoprecipitated with ANTI-FLAG M2 affinity gel, followed by western blot analysis with Flag and HA antibodies. $n=3$ independent experiments. **j,** SDS-PAGE analysis of the cell lysates with expression of vector, GST, Trx-MXT-1, GST-MTX-1, Trx-MXT-2, His-KLC-1-TPR, and GST-MIRO-1. $n=3$ independent experiments. Source data are provided as a Source Data file.

Supplementary Fig. 4

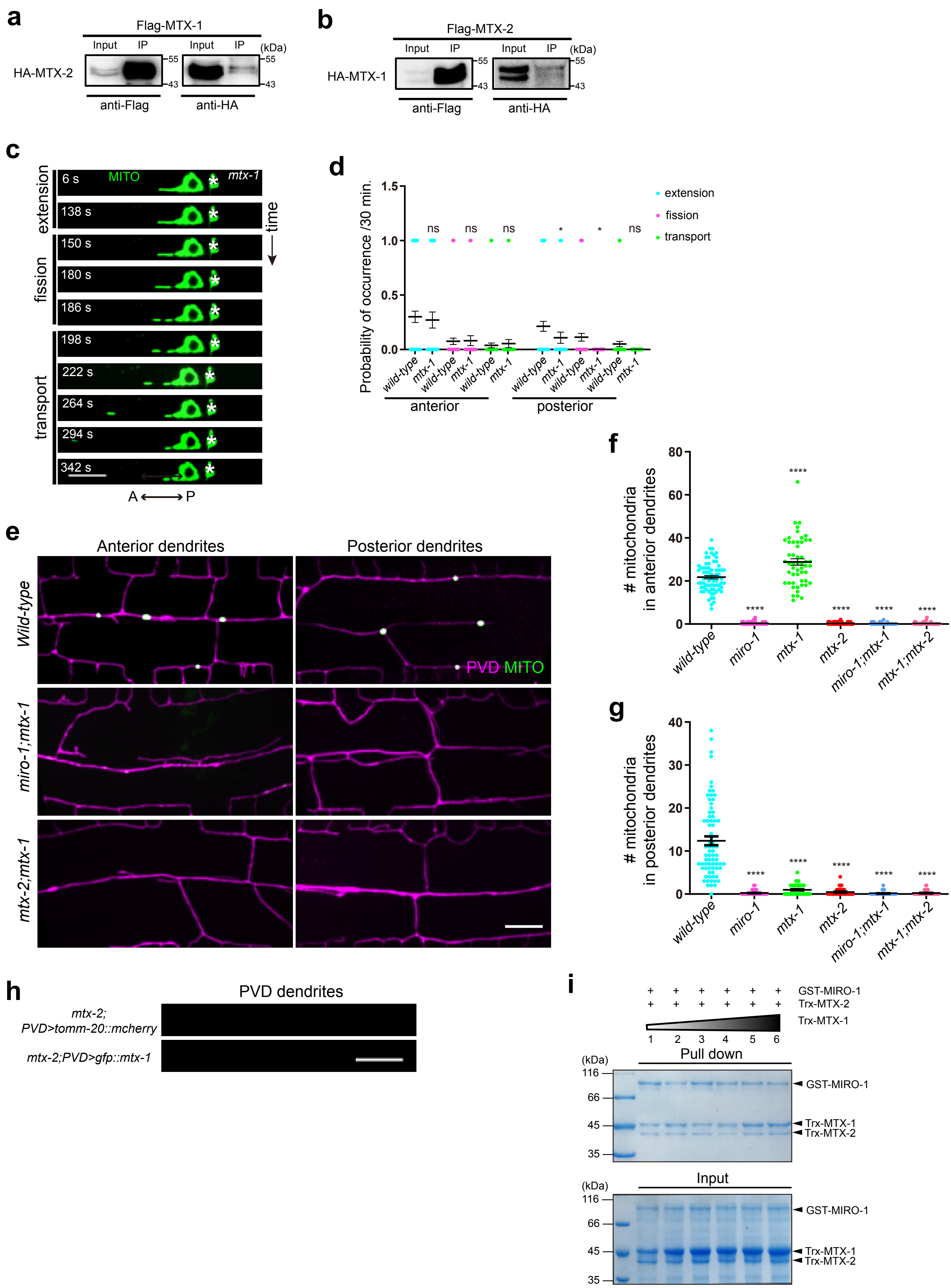

Supplementary Fig. 4: Genetic and biochemical interaction between metaxin complex and MIRO-1, related to Fig. 3, 4.

a, HA-MTX-2 were cotransfected with FLAG-MTX-1 in HEK293T cells, respectively, and then the cell lysates were immunoprecipitated with ANTI-FLAG M2 affinity gel, followed by western blot analysis with Flag and HA antibodies. *n*=3 independent experiments. **b,** HA-MTX-1 were cotransfected with FLAG-MTX-2 in HEK293T cells, respectively, and then the cell lysates were immunoprecipitated with ANTI-FLAG M2 affinity gel, followed by western blot analysis with Flag and HA antibodies. *n*=3 independent experiments. **c,** Representative time lapse imaging of mitochondria dynamics in dendrites of PVD in *mtx-1* mutant. Asterisk indicates PDE cell body. Scale bar: 10 μm. **d,** Quantification of mitochondrial dynamics in anterior and posterior dendrites of PVD in wild type and *mtx-1* mutant. Fisher's exact test was used. $p>0.05$, not significant, *$p<0.05$. $n>30$ 30-minute movies for each genotype. **e,** Representative confocal images showing dendritic morphology and mitochondrial distribution of PVD neuron in wild type, *miro-1 mtx-1,* and *mtx-2 mtx-1* double mutants. Magenta: PVD>mCherry. Green: PVD>TOMM-20 (1-54AA)::GFP. Scale bar: 10 μm. **f, g,** Quantification of mitochondria number in anterior (**f**) and posterior (**g**) dendrites in wild type, *miro-1, mtx-1, mtx-2, miro-1 mtx-1,* and *mtx-2 mtx-1* mutants. Data are shown as mean±SEM. One way ANOVA with Tukey's multiple comparisons test (95% CI). ****$p<0.0001$. (exact *p* values and sample size: **f,** *wild-type* vs. *miro-1*, $p<0.0001$; *wild-type* vs. *mtx-1*, $p<0.0001$; *wild-type* vs. *mtx-2*, $p<0.0001$; *wild-type* vs. *miro-1 mtx-1*, $p<0.0001$; *wild-type* vs. *mtx-1 mtx-2*, $p<0.0001$. *wild-type*, *n*=72; *miro-1*, *n*=72; *mtx-1*, *n*=52; *mtx-2*, *n*=72; *miro-1 mtx-1*, *n*=45; *mtx-1 mtx-2*, *n*=45 animals. **g,** *wild-type* vs. *miro-1*, $p<0.0001$; *wild-type* vs. *mtx-1*, $p<0.0001$; *wild-type* vs. *mtx-2*, $p<0.0001$; *wild-type* vs. *miro-1 mtx-1*, $p<0.0001$; *wild-type* vs. *mtx-1 mtx-2*, $p<0.0001$. *wild-type*, *n*=72; *miro-1*, *n*=72; *mtx-1*, *n*=60; *mtx-2*, *n*=72; *miro-1 mtx-1*, *n*=45; *mtx-1 mtx-2*, *n*=45 animals). **h,** Representative confocal images showing *mtx-2;* PVD>*tomm-20::mcherry* and *mtx-2;* PVD>*gfp::mtx-1* in PVD dendrites. Scale bar: 10 μm. **i,** GST pull-down assay performed using constant amount of purified GST-MIRO-1, and Trx-MTX-2, but the amount of purified Trx-MTX-1 was varied in each one (from lane 1 to 6: the amount of Trx-MTX-1 was increased with the ratio of 1.0, 2.2, 2.4, 2.6, 2.8, and 3.0, respectively). SDS-PAGE and Commassie blue staining of input and pull down material. Source data are provided as a Source Data file.

Supplementary Fig. 5

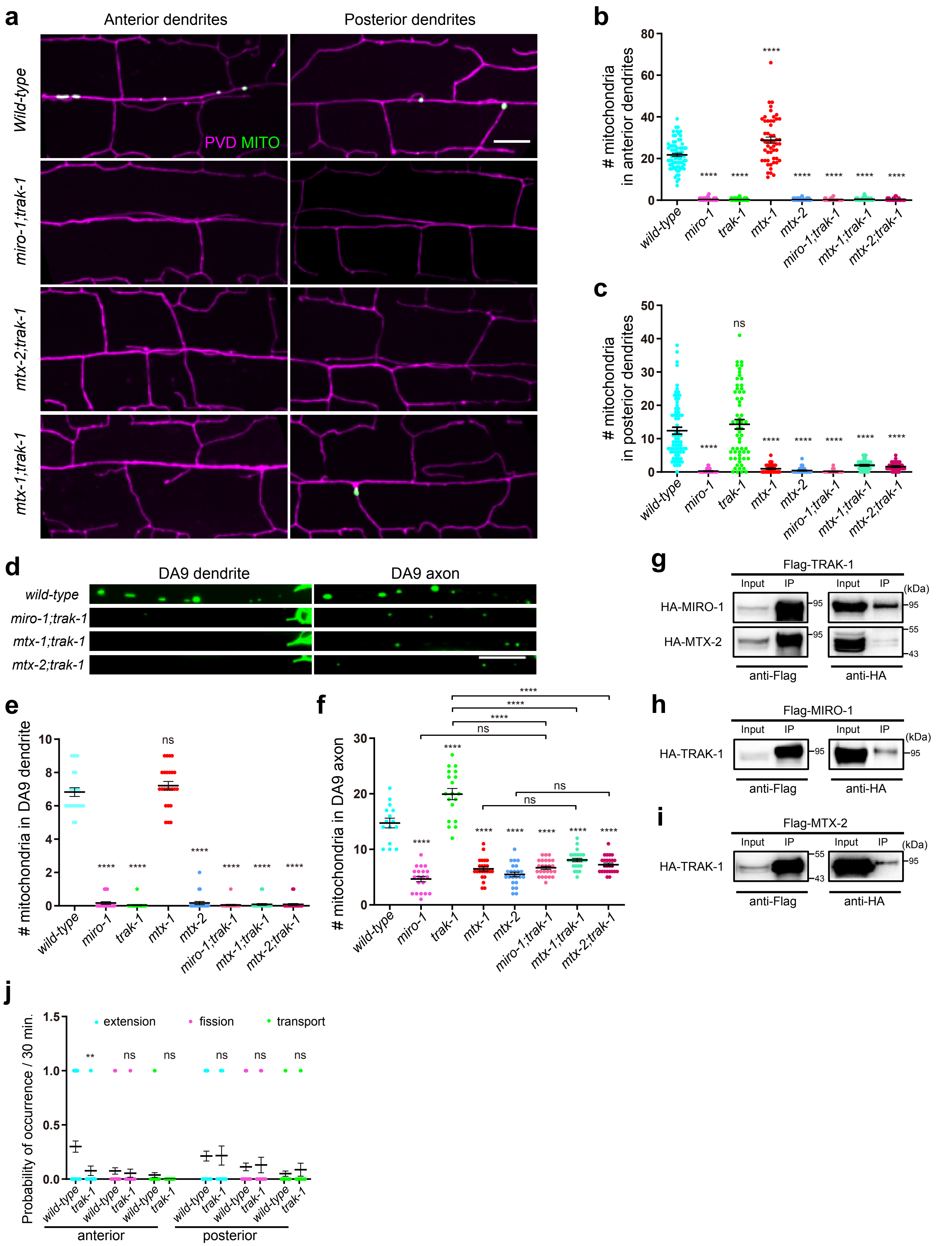

Supplementary Fig. 5: Genetic and biochemical interaction between the metaxin complex, MIRO-1, and TRAK-1, related to Fig. 5.

a, Representative confocal images showing dendritic morphology and mitochondrial distribution of PVD neuron in wild type, *miro-1 trak-1, mtx-2 trak-1,* and *mtx-1 trak-1* double mutants. Magenta: PVD>mCherry. Green: PVD>TOMM-20 (1-54AA)::GFP. Scale bar: 10 μm. **b, c,** Quantification of mitochondria number in anterior (**b**) and posterior (**c**) dendrites in wild type, *miro-1, trak-1, mtx-1, mtx-2, miro-1 trak-1, mtx-2 trak-1,* and *mtx-1 trak-1* mutants. Data are shown as mean±SEM. One way ANOVA with Tukey's multiple comparisons test (95% CI). $p>0.05$, not significant, ****$p<0.0001$. (exact p values and sample size: **b,** *wild-type* vs. *miro-1*, $p<0.0001$; *wild-type* vs. *trak-1*, $p<0.0001$; *wild-type* vs. *mtx-1*, $p<0.0001$; *wild-type* vs. *mtx-2*, $p<0.0001$; *wild-type* vs. *miro-1 trak-1*, $p<0.0001$; *wild-type* vs. *mtx-1 trak-1*, $p<0.0001$. *wild-type* vs. *mtx-2 trak-1*, $p<0.0001$. *wild-type*, $n=72$; *miro-1*, $n=72$; *trak-1*, $n=72$, *mtx-1*, $n=52$; *mtx-2*, $n=72$; *miro-1 trak-1*, $n=45$; *mtx-1 trak-1*, $n=45$; *mtx-2 trak-1*, $n=45$ animals. **c,** *wild-type* vs. *miro-1*, $p<0.0001$; *wild-type* vs. *trak-1*, $p=0.4393$; *wild-type* vs. *mtx-1*, $p<0.0001$; *wild-type* vs. *mtx-2*, $p<0.0001$; *wild-type* vs. *miro-1 trak-1*, $p<0.0001$; *wild-type* vs. *mtx-1 trak-1*, $p<0.0001$. *wild-type* vs. *mtx-2 trak-1*, $p<0.0001$. *wild-type*, $n=72$; *miro-1*, $n=72$; *trak-1*, $n=60$, *mtx-1*, $n=60$; *mtx-2*, $n=72$; *miro-1 trak-1*, $n=45$; *mtx-1 trak-1*, $n=45$; *mtx-2 trak-1*, $n=45$ animals). **d,** Representative confocal images showing mitochondrial distribution of DA9 neuron in wild type, *miro-1 trak-1, mtx-1 trak-1,* and *mtx-2 trak-1* double mutants. Green: DA9>TOMM-20 (1-54AA)::GFP. Scale bar: 10 μm. **e, f,** Quantification of mitochondria number in dendrite and axon of DA9 in wild type, *miro-1, trak-1, mtx-1, mtx-2, miro-1 trak-1, mtx-1 trak-1,* and *mtx-2 trak-1* mutants. Data are shown as mean±SEM. One way ANOVA with Tukey's multiple comparisons test (95% CI). $p>0.05$, not significant, ****$p<0.0001$. (exact p values and sample size: **e,** *wild-type* vs. *miro-1*, $p<0.0001$; *wild-type* vs. *trak-1*, $p<0.0001$; *wild-type* vs. *mtx-1*, $p=0.5273$; *wild-type* vs. *mtx-2*, $p<0.0001$; *wild-type* vs. *miro-1 trak-1*, $p<0.0001$; *wild-type* vs. *mtx-1 trak-1*, $p<0.0001$; *wild-type* vs. *mtx-2 trak-1*, $p<0.0001$. *wild-type*, $n=23$; *miro-1*, $n=24$; *trak-1*, $n=28$; *mtx-1*, $n=24$; *mtx-2*, $n=24$; *miro-1 trak-1*, $n=24$; *mtx-1 trak-1*, $n=24$; *mtx-2 trak-1*, $n=24$ animals. **f,** *wild-type* vs. *miro-1*, $p<0.0001$; *wild-type* vs. *trak-1*, $p<0.0001$; *wild-type* vs. *mtx-1*, $p<0.0001$; *wild-type* vs. *mtx-2*, $p<0.0001$; *wild-type* vs. *miro-1 trak-1*, $p<0.0001$; *wild-type* vs. *mtx-1 trak-1*, $p<0.0001$; *wild-type* vs. *mtx-2 trak-1*, $p<0.0001$; *miro-1* vs. *miro-1 trak-1*, $p=0.0859$; *trak-1* vs. *miro-1 trak-1*, $p<0.0001$; *trak-1* vs. *mtx-1 trak-1*, $p<0.0001$; *trak-1* vs. *mtx-2 trak-1*, $p<0.0001$; *mtx-1* vs. *mtx-1 trak-1*, $p=0.2736$; *mtx-2* vs. *mtx-2 trak-1*, $p=0.1691$. *wild-type*, $n=15$; *miro-1*, $n=20$; *trak-1*, $n=19$; *mtx-1*, $n=23$; *mtx-2*, $n=23$; *miro-1 trak-1*, $n=25$; *mtx-1 trak-1*, $n=25$; *mtx-2 trak-1*, $n=25$ animals). **g,** HA-MIRO-1 or HA-MTX-2 were cotransfected with FLAG-TRAK-1 in HEK293T cells, respectively, and then the cell lysates were immunoprecipitated with ANTI-FLAG M2 affinity gel, followed by western blot analysis with Flag and HA antibodies. $n=3$ independent experiments. **h, i,** FLAG-MIRO-1 or FLAG-MTX-2 were cotransfected with HA-TRAK-1 in HEK293T cells, respectively, and then the cell lysates were immunoprecipitated with ANTI-FLAG M2 affinity gel, followed by western blot analysis with Flag and HA antibodies. $n=3$ independent experiments. **j,** Quantification of mitochondrial dynamics in anterior and posterior dendrites of PVD in wild type and *trak-1* mutant. Fisher's exact test was used. $p>0.05$, not significant, **$p<0.01$. $n>30$ 30 minute movies for each genotype. Source data are provided as a Source Data file.

Supplementary Fig. 6

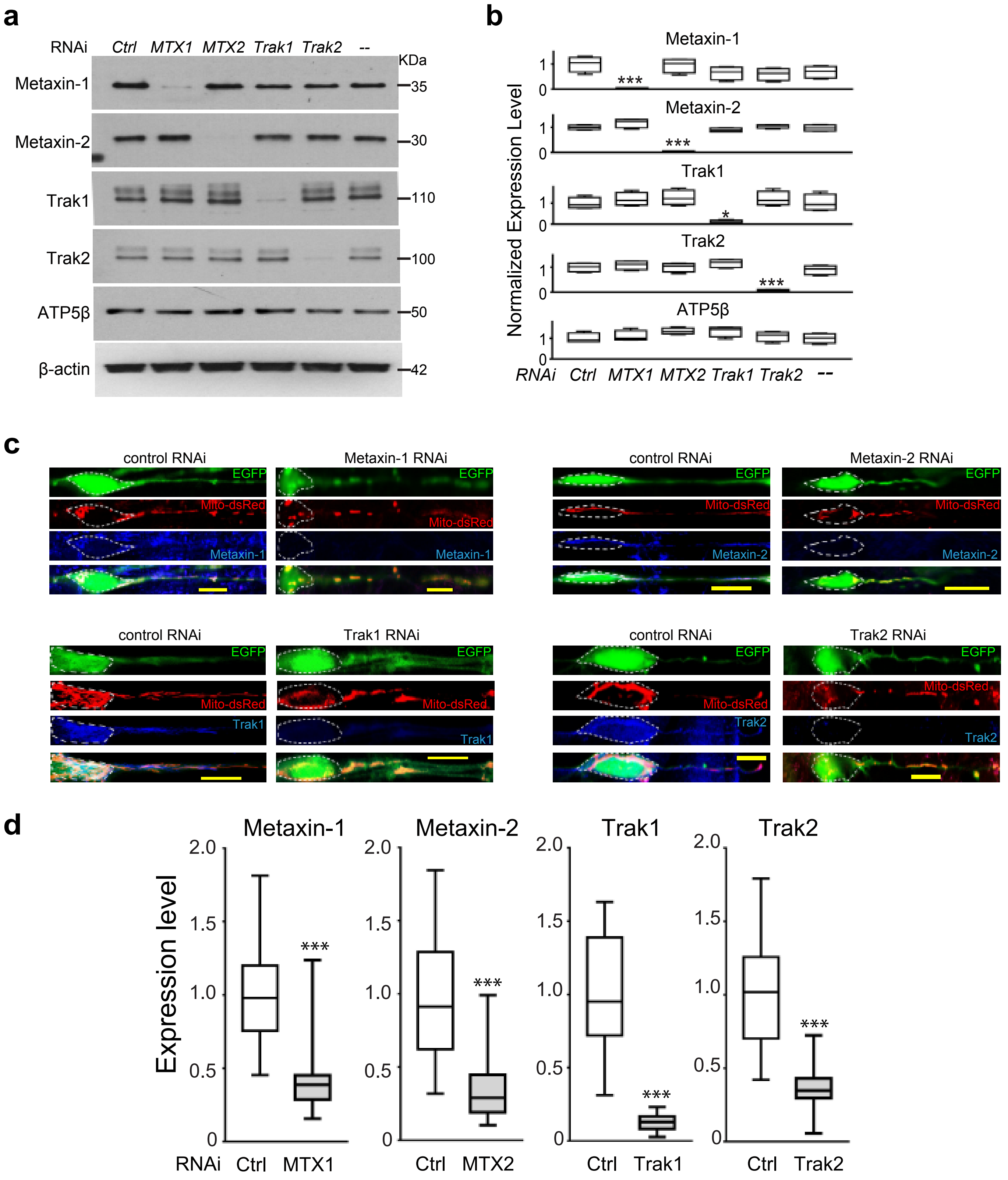

Supplementary Fig. 6: Efficiency of RNAi, related to Fig. 7.

a, HEK293T cells were transfected with RNAi, lysed, and immunoblotted as indicated. **b,** The intensity of each band is normalized to that of the loading control β-actin from the same blot, and expressed as a fraction of the mean value of "control RNAi". Data are shown as box plots with with minimum, median, maximum and lower-upper interquartile range. One-way ANOVA with Tukey's multiple comparisons test (95% CI). *$p<0.05$, **$p<0.01$, ***$p<0.001$. $n=4$ independent experiments. The band intensities of β-actin are not significantly different among all conditions ($p=0.9876$). **c,** iPSC-derived neurons were transfected with EGFP (green), mito-dsRed (red) and RNAi as indicated at day 20 after neural induction, and immunostained 3 days later with antibodies as indicated (blue). Scale bars: 10 μm. **d,** Quantification of the fluorescent intensity of the indicated protein (blue) normalized to that of mito-dsRed (red) in the same neuronal cell body also positive for EGFP, exampled with white dashed lines in (**c**). Compared with the data point of control RNAi in the same immunostaining group. $n=29\text{-}70$ cell bodies from 2 independent transfections. The intensities of EGFP and mito-dsRed are not significantly different among all conditions ($p>0.2$). Two-tailed unpaired *t*-test (95% CI). *$p<0.05$, **$p<0.01$, ***$p<0.001$. $p<0.0001$ (Metaxin-1), $p<0.0001$ (Metaxin-2), $p<0.0001$ (Trak1), and $p<0.0001$ (Trak2). $n=56$ (Ctrl RNAi), 34 (*MTX1* RNAi) (**Metaxin-1**), 49 (Ctrl RNAi), 34 (*MTX2* RNAi) (**Metaxin-2**), 29 (Ctrl RNAi), 33 (*Trak1* RNAi) (**Trak1**), and 69 (Ctrl RNAi), 70 (*Trak2* RNAi) (**Trak2**) from 2 independent transfections. Source data are provided as a Source Data file.

Supplementary Table 1: Strains used in this study

Strain name	Genotype	Source	Notes/Purpose
CU6372	*drp-1(tm1108)*	CGC	composite in/del
EV828	*dhc-1(or195ts)*	CGC	weak allele
FF41	*unc-116(e2310)*	CGC	weak allele
KU801	*klc-2(km11)*	CGC	large fragment deletion
RB1975	*klc-1(ok2609)*	CGC	large fragment deletion
TV15911	*wyIs592*	Integration	to visualize and study PVD(gfp) neuron
TV15921	*wyIs594*	Integration	to visualize and study PVD(gfp) neuron
TV50356	*miro-1(wy50180)*	made by Cas9	7 bp deletion in the first exon
TV50371	*trak-1(wy50182);wyIs592*	crossing	to visualize and study PVD branches in *trak-1* mutant
TV50372	*miro-1(wy50180);wyIs592*	crossing	to visualize and study PVD branches in *miro-1* mutant
TV50426	*dli-1(wy50053)*	made by EMS	premature stop codon
TV50671	*dli-1(wy50053);wyIs50054*	crossing	to visualize and study mitochondria distribution and dynamics of *dli-1* mutant in PVD
TV50673	*drp-1(tm1108);wyIs50054*	crossing	to visualize and study mitochondria morphology, distribution and dynamics of *drp-1* mutant in PVD
TV50680	*klc-2(km11);wyIs50054*	crossing	to visualize and study mitochondria distribution and dynamics of *klc-2* mutant in PVD
TV50684	*miro-1(wy50180);wyIs50054*	crossing	to visualize and study mitochondria distribution and dynamics of *miro-1* mutant in PVD
TV50692	*trak-1(wy50182);wyIs50054*	crossing	to visualize and study mitochondria distribution and dynamics of *trak-1* mutant in PVD
TV50695	*unc-116(e2310);wyIs50054*	crossing	to visualize and study mitochondria distribution and dynamics of *unc-116* mutant in PVD
TV50712	*trak-1(wy50182);miro-1(wy50180);wyIs50054*	crossing	to visualize and study mitochondria distribution and dynamics of *trak-1;miro-1* double mutant in PVD
TV50713	*trak-1(wy50182);dli-1(wy50235);wyIs50054*	crossing	to visualize and study mitochondria distribution and dynamics of *trak-1;dli-1* double mutant in PVD
TV51425	*miro-1(wy50285 [gfp::miro-1])*	made by	gfp knockin in *miro-1* N-terminal for

		Cas9	monitoring endogenous *miro-1* expression level
TV51426	*miro-1(wy50260)*	made by EMS	237 bp deletion
TV51427	*miro-1(wy50233)*	made by EMS	mutation at a splice site
TV51428	*trak-1(wy50182)*	made by Cas9	premature stop codon in second exon
TV51429	*mtx-1(wy50272)*	made by Cas9	5 bp deletion in the second exon
TV51430	*mtx-1(wy50286)*	made by Cas9	composite in/del
TV51431	*mtx-2(wy50250);wyIs50054*	crossing	to visualize and study mitochondria distribution and dynamics of *mtx-2* weak allele in PVD
TV51432	*mtx-2(wy50266)*	made by Cas9	22 bp deletion in the first exon
TV51433	*mtx-2(wy50250)*	made by EMS	V144M
TV51434	*mtx-2(wy50256)*	made by EMS	Q9X
TV51435	*mtx-2(wy50266);wyIs50054*	crossing	to visualize and study mitochondria distribution and dynamics of *mtx-2* null allele in PVD
TV51436	*mtx-2(gk444);wyIs50054*	crossing	to visualize and study mitochondria distribution and dynamics of *mtx-2* null allele in PVD
TV51437	*miro-1(wy50260);wyIs50054*	crossing	to visualize and study mitochondria distribution and dynamics of *miro-1* mutant in PVD
TV51438	*mtx-1(wy50286);wyIs50054*	crossing	to visualize and study mitochondria distribution and dynamics of *mtx-1* null allele in PVD
TV51439	*miro-1(wy50233);wyIs50054*	crossing	to visualize and study mitochondria distribution and dynamics of *miro-1* mutant in PVD
TV51440	*dli-1(wy50235)*	made by EMS	premature stop codon in the last exon
TV51441	*mtx-1(wy50272);wyIs50054*	crossing	to visualize and study mitochondria distribution and dynamics of *mtx-1* null allele in PVD
TV51442	*wySi50001;unc-119(ed4)*	made by miniMos	*PVD>gfp::mtx-2*
TV51443	*wySi50002;unc-119(ed4)*	made by	*PVD>gfp::mtx-1*

		miniMos	
TV51444	*wySi50004;unc-119(ed4)*	made by miniMos	*PVD>miro-1*
TV51445	*mtx-1(ok3155);wyIs50054*	crossing	to visualize and study mitochondria distribution and dynamics of *mtx-1* mutant in PVD
TV51446	*mtx-2(wy50266);wyIs594*	crossing	to visualize and study PVD branches in *mtx-2* null allele
TV51447	*wyIs50054*	Integration	to visualize and study PVD branches(mCherry) and mitochondria(gfp) in PVD
TV51448	*wyIs50091*	Integration	to visualize and study mitochondria(gfp) and synapse(mCherry) in DA9
TV51449	*miro-1(wy50180);wyIs50054;wySi50004;unc-119(ed4)*	crossing	
TV51450	*trak-1(wy50182);wyIs50054;wySi50004;unc-119(ed4)*	crossing	
TV51451	*trak-1(wy50182);mtx-2(wy50266);wyIs50054*	crossing	to visualize and study mitochondria distribution and dynamics of *trak-1;mtx-2* double mutant in PVD
TV51452	*trak-1(wy50182);mtx-1(wy50272);wyIs50054*	crossing	to visualize and study mitochondria distribution and dynamics of *trak-1;mtx-1* double mutant in PVD
TV51453	*mtx-1(wy50272);miro-1(wy50180);wyIs50054*	crossing	to visualize and study mitochondria distribution and dynamics of *mtx-1; miro-1* double mutant in PVD
TV51454	*mtx-2(wy50266);miro-1(wy50180);wyIs50054*	crossing	to visualize and study mitochondria distribution and dynamics of *mtx-2;miro-1* double mutant in PVD
TV51455	*mtx-2(wy50266);mtx-1(wy50272);wyIs50054*	crossing	to visualize and study mitochondria distribution and dynamics of *mtx-1;mtx-2* double mutant in PVD
TV51456	*mtx-2(wy50266);wyIs50054;wySi50004;unc-119(ed4)*	crossing	
TV51457	*dli-1(wy50235);wyIs50054*	crossing	to visualize and study mitochondria distribution and dynamics of *dli-1* mutant in PVD
TV51458	*klc-1(ok2609);wyIs50054*	crossing	to visualize and study mitochondria distribution and dynamics of *klc-1* mutant in PVD
TV51460	*trak-1(wy50182);wySi50001;unc-119(ed4)*	crossing	
TV51461	*trak-1(wy50182);wySi50002;unc-119(ed4)*	crossing	

TV51462	*miro-1(wy50180);wySi50001;unc-119(ed 4)*	crossing	
TV51463	*mtx-1(wy50266);wySi50002;unc-119(ed 4)*	crossing	
TV51464	*wyEx50750*	injection	*ser-2*P3::*vdac-1::gfp*(5ng/ul); *ser-2*P3::*myri-mcherry*(10ng/ul); P*ord-1*>*gfp*(30ng/ul);N2
TV51465	*mtx-1(wy50272);wySi50001;unc-119(ed 4)*	crossing	
TV51466	*mtx-1(wy50272);wyIs50091*	crossing	to visualize and study mitochondria distribution and dynamics of *mtx-1* mutant in DA9
TV51467	*mtx-1(wy50272);miro-1(wy50285 [gfp::miro-1])*	crossing	Monitoring endogenous *miro-1* expression level in *mtx-1* mutant
TV51468	*mtx-2(wy50266);wySi50001;unc-119(ed 4)*	crossing	
TV51469	*mtx-2(wy50266);wyIs50091*	crossing	to visualize and study mitochondria distribution and dynamics of *mtx-2* mutant in DA9
TV51470	*mtx-2(wy50266);miro-1(wy50285 [gfp::miro-1])*	crossing	Monitoring endogenous *miro-1* expression level in *mtx-2* mutant
TV51471	*mtx-2(wy50266);miro-1(wy50180);wyIs50091*	crossing	to visualize and study mitochondria distribution and dynamics of *mtx-2;miro-1* double mutant in DA9
TV51472	*mtx-1(wy50272);wyIs592*	crossing	to visualize and study PVD branches in *mtx-1* mutant
TV51473	*mtx-2(wy50266);wySi50002;unc-119(ed 4)*	crossing	
TV51474	*klc-2(km11);wyIs50091*	crossing	to visualize and study mitochondria distribution and dynamics of *klc-2* mutant in DA9
TV51475	*miro-1(wy50180);wyIs50091*	crossing	to visualize and study mitochondria distribution and dynamics of *miro-1* mutant in DA9
TV51476	*trak-1(wy50182);wyIs50091*	crossing	to visualize and study mitochondria distribution and dynamics of *trak-1* mutant in DA9
TV51477	*unc-116(e2310);wyIs50091*	crossing	to visualize and study mitochondria distribution and dynamics of *unc-116* mutant in DA9
TV51478	*trak-1(wy50182);miro-1(wy50180);wyIs 50091*	crossing	to visualize and study mitochondria distribution and dynamics of *unc-116* mutant in DA9
TV51479	*mtx-1(wy50272);miro-1(wy50180);wyIs5*	crossing	to visualize and study mitochondria

	0091		distribution and dynamics of *mtx-1;miro-1* double mutant in DA9
TV51480	*trak-1(wy50182);mtx-1(wy50272);wyIs50091*	crossing	to visualize and study mitochondria distribution and dynamics of *trak-1;mtx-1* double mutant in DA9
TV51481	*trak-1(wy50182);mtx-2(wy50266);wyIs50091*	crossing	to visualize and study mitochondria distribution and dynamics of *trak-1;mtx-2* double mutant in DA9
TV51482	*mtx-2(wy50266);miro-1(wy50180);mtx-1(wy50272);wyIs50091*	crossing	to visualize and study mitochondria distribution and dynamics of *miro-1;mtx-1;mtx-2* triple mutant in DA9
TV51483	*mtx-1(wy50272);wySi50002;unc-119(ed4)*	crossing	
TV51484	*miro-1(wy50180);wySi50002;unc-119(ed4)*	crossing	
TV51485	*mtx-2(wy50266);mtx-1(wy50272);wyIs50091*	crossing	to visualize and study mitochondria distribution and dynamics of *mtx-1;mtx-2* double mutant in DA9
TV51487	*dli-1(wy50235);wyIs50091*	crossing	to visualize and study mitochondria distribution and dynamics of *dli-1* mutant in DA9
TV51488	*klc-1(ok2609);wyIs50091*	crossing	to visualize and study mitochondria distribution and dynamics of *klc-1* mutant in DA9
TV51489	*wyEx50521*	injection	*ser-2*Prom3>*fzo-1::gfp*(10ng/ul); *ser-2*Prom3>*tomm-20(1-54aa)::mCherry* (10ng/ul); P*ord-1*>*gfp*(30ng/ul); N2
TV51490	*wyEx50801*	injection	P*sepa-1*>tomm-20(1-54aa)::mCherry (5ng/ul); N2
TV51491	*wyIs50082*	Integration	to visualize and study mitochondria(mCherry) in PVD
TV51492	*wyIs50082;wySi50001;unc-119(ed4)*	crossing	
TV51493	*wyIs50082;wySi50002;unc-119(ed4)*	crossing	
TV51494	*wyEx50801;miro-1(wy50285 [gfp::miro-1])*	crossing	confirming *miro-1* subcellular localization
TV51495	*wyIs50082;wySi50002;mtx-2(wy50266); unc-119(ed4)*	crossing	
TV51496	*wyEx50802*	injection	*PVD*>unc-116 cDNA (100ng/ul); *PVD*>unc-116 cDNA complement (100ng/ul); P*myo-2*>mcherry(1ng/ul); wyIs50054
TV51497	*mtx-2(wy50256);wyIs50054*	crossing	to visualize and study mitochondria distribution and dynamics of *mtx-2* null allele in PVD

TV51811	*wySi50085;unc-119(ed4)*	made by miniMos	*PVD*>*gfp::rab-5* (early endosome marker)
TV51812	*wySi50086;unc-119(ed4)*	made by miniMos	*PVD*>*gfp::rab-7* (late endosome marker)
TV51813	*wySi50085;unc-119(ed4);miro-1(wy50180)*	crossing	
TV51814	*wySi50086;unc-119(ed4);miro-1(wy50180)*	crossing	
TV51815	*wySi50085;unc-119(ed4);mtx-2(wy50266)*	crossing	
TV51816	*wySi50086;unc-119(ed4);mtx-2(wy50266)*	crossing	
TV51817	*gop-3(tm3269)/hT2 [qIs48];wyIs50054*	crossing	
TV51818	*gop-3(tm3269)/hT2 [qIs48];wySi50001;unc-119(ed4)*	crossing	
TV51819	*wyIs50082;gop-3(tm3269)/hT2 [qIs48];wySi50001;unc-119(ed4)*	crossing	
VC1064	*mtx-2(gk444)*	CGC	525 bp deletion
VC2415	*mtx-1(ok3155)*	CGC	371 bp deletion

Supplementary Table 2: Plasmids used in this study

Plasmid name	Detailed information	Purpose
pOL036	ser-2prom3::myri-mCherry	worm genetics
pXM222	pPD95.77_ser-2Prom3::tomm-20(1-54aa)::mCherry	worm genetics
pXM250	pPD95.77_ser-2Prom3::fzo-1::gfp	worm genetics
pXM271	Peft-3_miro-1_gRNA	worm genetics
pXM273	Peft-3_trak-1_gRNA	worm genetics
pXM289	pPD95.77_ser-2Prom3::tomm-20(1-54aa)::gfp	worm genetics
pXM452	pPD95.77_ser-2Prom3::vdac-1::gfp	worm genetics
pYS109	ser-2P3::gfp::mtx-2::unc-54_3'UTR SCI plasmid	worm genetics
pYS120	ser-2P3::gfp::mtx-1::unc-54_3'UTR SCI plasmid	worm genetics
pYS129	Peft-3_miro-1_gRNA 1	worm genetics
pYS130	Peft-3_miro-1_gRNA 2	worm genetics
pYS131	pPD95.77_homo_gfp::miro-1	worm genetics
pYS136	Peft-3_miro-1_gRNA 3	worm genetics
pYS137	pPD95.77_homo_gfp::miro-1(base sub.in first exon)	worm genetics
pYS138	pPD95.77_homo_gfp::miro-1(base sub.in first exon)	worm genetics
pYS139	Peft-3_miro-1_gRNA 4	worm genetics
pYS140	Peft-3_miro-1_gRNA 5	worm genetics
pYS36	pPD95.77_ser-2P3::gfp::miro-1	worm genetics
pYS379	pPD95.77_ser-2P3::unc-116 fragment_F(1400 bp)	worm genetics
pYS38	pPD95.77_ser-2P3::mtx-2	worm genetics
pYS40	pPD95.77_ser-2P3::mtx-1	worm genetics
pYS402	pPD95.77_ser-2P3::unc-116 fragment_R(1400 bp)	worm genetics
pYS410	pPD95.77_Psepa-1_tomm-20(1-54aa)::mcherry	worm genetics
pYS436	ser-2P3::miro-1::unc-54_3'UTR SCI plasmids	worm genetics
pYS526	ser-2P3::GFP::rab-5(cDNA)::unc-54_3'UTR SCI plasmid	worm genetics
pYS527	ser-2P3::GFP::rab-7(cDNA)::unc-54_3'UTR SCI plasmid	worm genetics
pYS56	pPD95.77_ser-2P3::gfp::mtx-1	worm genetics
pYS57	pPD95.77_ser-2P3::gfp::mtx-2	worm genetics
pYS59	Peft-3_mtx-2_gRNA 1	worm genetics
pYS60	Peft-3_mtx-2_gRNA 2	worm genetics
pYS79	pPD95.77_ser-2P3::miro-1	worm genetics
pYS86	Peft-3_mtx-1_gRNA 1	worm genetics

pYS87	Peft-3_mtx-1_gRNA 2	worm genetics
pYS88	Peft-3_mtx-1_gRNA 3	worm genetics
pYS163	pGEX-6P-1_mtx-1(de.TM)	gel-filtration and GST pull-down assay
pYS186	pGEX-6P-1_miro-1(de.TM)	gel-filtration and GST pull-down assay
pYS190	pET.32M.3C_mtx-1(de.TM)	gel-filtration and GST pull-down assay
pYS191	pET.32M.3C_mtx-2	gel-filtration and GST pull-down assay
pYS213	pET.M.3C_miro-1(de.TM)	gel-filtration and GST pull-down assay
pYS250	pRSFDuet-1_Trx_mtx-2(MCS 1)_Trx_mtx-1(de.TM)(MCS 2)	gel-filtration and GST pull-down assay
pYS251	pRSFDuet-1_miro-1(de.TM)(MCS 1)_Trx_mtx-2(MCS 2)	gel-filtration and GST pull-down assay
pYS254	pRSFDuet-1_Trx_mtx-2(MCS 1)_GST_mtx-1(de.TM)(MCS 2)	gel-filtration and GST pull-down assay
pYS339	pET.M.3C_klc-1_TPR domain	gel-filtration and GST pull-down assay
pYS372	pRSFDuet-1_Trx_mtx-2(MCS 1)_GST(MCS 2)	gel-filtration and GST pull-down assay
pYS110	pNTAP-B_3*Flag	Co-IP assay
pYS111	pNTAP-B_3*Flag_miro-1	Co-IP assay
pYS112	pNTAP-B_3*Flag_mtx-1	Co-IP assay
pYS113	pNTAP-B_3*Flag_mtx-2	Co-IP assay
pYS114	pNTAP-B_3*Flag_trak-1	Co-IP assay
pYS115	pNTAP-B_3*HA_miro-1	Co-IP assay
pYS116	pNTAP-B_3*HA_mtx-1	Co-IP assay
pYS117	pNTAP-B_3*HA_mtx-2	Co-IP assay
pYS175	pNTAP-B_3*Flag_drp-1	Co-IP assay

Supplementary Table 3: Primer used for genotyping

Primer name	Primer Sequence (5' ---> 3')
miro-1 gRNA check F	TGAGCGACGACGAGACGTTG
miro-1 gRNA check R	TCACCAGCGATGTCTTTCCG
miro-1 mutant check F	cacggaaacgctctgtcagtt
miro-1 mutant check R	TCCACGATGCTCGTCGTGACG
miro-1(wy50180) check R	TCAGAACGATTCGGACGT
miro-1 seq. 1R	catcagatctggcctcaa
miro-1 seq. 2F	ttgaggccagatctgatg
miro-1 seq. 2R	gtaacaaatcctagttgg
miro-1 seq 1Fs	CAGATGGATGCCCAGATG
miro-1(wy50229) check R	CTGGCTTCTGACACACGA
miro-1 CDS seq. F	GTGGCTACCCCTGATTCG
miro-1_GFP knock in check F1	ggatccgagttgtagcatg
miro-1_GFP knock in check F2	ccgtttctgtcgagagctt
miro-1_GFP knock in check R1	GAAAGTAGTGACAAGTGTTGGct
miro-1 CDS seq. F2	TATGGCATATTGGAACATG
miro-1(tm1966) check inner F	ATGATTCTCTGATGCTCGCC
trak-1 gRNA check F	GAGCCCAACTCCAACTCGAAAC
trak-1 gRNA check R	TAACCATGAGACCATTGCAG
trak-1 mutant check F	agtgccgcccgtccgctt
trak-1 mutant check R	CAAGCTCAAGATCGTGAT
trak-1(wy50182) check R	ATCAAGTGGAGAGGAGTT
trak-1 seq. 1F	tattcggatttataagga
trak-1 seq. 1R	gccacgcttgatataaat
trak-1 seq. 2F	atttatatcaagcgtggc
trak-1 seq. 2R	acCTATATGCAACCGTTG
trak-1 seq. 3F	ATTCCCTCGATCGCAGAG
trak-1 seq. 3R	gtggagtgggggtagaac
trak-1 seq 1Fs	agaacgtttccgcatcat
trak-1 seq 3Rs	aacgtcgcgtttgctgat
trak-1(wy50182) check new R	GCTAGAAAGTTTCGAGTT
trak-1(tm1572) check outer F	ATCACGATCTTGAGCTTG
mtx-1(ok3155) check out F	atggccgtgaccgaggtt
mtx-1(ok3155) check out/inner R	AATTGAGCTCGTCCGTCC
mtx-1(ok3155) check inner F	CCAACTATTGACGTTGTGTC
mtx-2(wy50267) check R	TTCAGAAGTGGGACGACTC
mtx-1(wy50272) check F	GCTCTAAAATGTGTGCCAGC
mtx-1 seq. F	TCCGCAGCTTCCGTGATTC
mtx-1 seq. R	CTCGTGGGTGGCAGTAGGTA
mtx-2 seq. F1	AAGCAACTGTCTCGTCACAAG
mtx-2 seq. R1	CACAATCAGGGATGGGACTTAT

mtx-2 seq. F2	CATCCCTGATTGTGCCACG
mtx-2 seq. R2	CCAGAAGTGATGACAACGACAT
mtx-2(wy50250) check F	aaactaacgttggtaaaaatttag
mtx-2(wy50250) check R	tacCTTGGTGTCCCAGTC
mtx-2(gk444) check F	CATCACAGGGTTCAACGC
mtx-2 cas9 mutant check F	gacgaccgagtgattctatt
mtx-2 cas9 mutant check R	GTTGACGAACATTGAATGGC
mtx-2(wy50267) check F	GATAACCCAATTGGTCACTG
unc-116_1F	gcattgtaaggagaagcc
unc-116_1R	TGAAAGTTCCAAAGCCGC
unc-116_2F	GCAGAAGAATGGAAACGG
unc-116_2R	cagtgtattgattggatc
unc-116 F	ATGCTGAAGCGAAACTTGC
unc-116 R	GCAGTTCAACGCGCAAATC
klc-1(ok2609) check outer/inner F	TTGGGTGACGATATCACA
klc-1(ok2609) check outer R	GCTTACGATAAACTGCTG
klc-1(ok2609) check inner R	CGGAGGTAGTGTGTTGct
klc-2(km11) outer F	agCAAAGTGATACTAAGA
klc-2(km11) outer R	AGTTGAATCCGAGTGCAT
klc-2(km11) inner R	TCGGGTCATCAGGTCCAA
klc-2(km11) inner_new_F	gcctgaacaatgtcttgc
klc-2(km11) outer_new_F	ggcagggctgttatttaagacg
dli-1 1 F	CAGACGAACTTCTCCGAGGGAC
dli-1 1 R	CCAACTACGGAAGTGCTCATT
dli-1 2 F	AGAAGACTTGAAAGCCGGTAG
dli-1 2 R	GATTCGTCGCCGCTTTCA
dli-1(wy50235) check F	GAGTCTATTCCGGATGTGGA
gop-3(tm3269) check outer F	cacgacctgaacaaagtcg
gop-3(tm3269) check outer/inner R	GAGTCGCTGATACATTCGA
gop-3(tm3269) check inner F	ACCATCCGTGCAAAGGCAT

Supplementary Table 4: Primer used for plasmid construction

Plasmids	Primer Sequence (5' ---> 3')
pXM222	tatgtgttgtgatgtcac
	GGTAGTGGAAGCGGCTCTATGGTCTCAAAGGGTGAAG
	acatcacaacacataATGTCGGACACAATTCTTGG
	GCCGCTTCCACTACCTCCAGCCTGGGCACGTCTCT
pXM250	tatgtgttgtgatgtcac
	GGTAGTGGAAGCGGCTCT
	acatcacaacacataATGTCTGGCACAGCAAGC
	GCCGCTTCCACTACCTGGCGTTGGCGGAGAGTC
pXM289	tatgtgttgtgatgtcac
	GGTAGTGGAAGCGGCTCTatgagtaaaggagaagaac
	acatcacaacacataATGTCGGACACAATTCTTGG
	GCCGCTTCCACTACCTCCAGCCTGGGCACGTCTCT
pXM452	tatgtgttgtgatgtcac
	GGTAGTGGAAGCGGCTCTatgagtaaaggagaagaac
	acatcacaacacataATGGCCCCACCAACCTTC
	GCCGCTTCCACTACCGTTGGATGGATCGAATTCGAG
pXM271	ACGTCCGAATCGTTCTGATGTTTTAGAGCTAGAAATAGC
	GAACGATTCGGACGTCaagacatctcgcaataggaggtg
pXM273	AGAGCACACGACGGATCAAGGTTTTAGAGCTAGAAATAGC
	TCCGTCGTGTGCTCTCaagacatctcgcaataggaggtg
pYS109	gtcgacagcgtttcgTGGCGTAATCATGGTCATACTAGT
	ACAGCTTGTCTGTAAGTACCCTCTAGTCAAGGCCT
	cgaaacgctgtcgacttcaac
	TTACAGACAAGCTGTGACCG
pYS120	gtcgacagcgtttcgTGGCGTAATCATGGTCATACTAGT
	ACAGCTTGTCTGTAAGTACCCTCTAGTCAAGGCCT
	cgaaacgctgtcgacttcaac
	TTACAGACAAGCTGTGACCG
pYS129	gaaagctcaagttcgagaagGTTTTAGAGCTAGAAATAGC
	cgaacttgagctttcCaagacatctcgcaataggaggtg
pYS130	aaaatcgtataaaacggaggGTTTTAGAGCTAGAAATAGC
	gttttatacgattttCaagacatctcgcaataggaggtg
pYS131	TTTTTCTACCGGTACCCTCAAGGG
	ATGAGTAAAGGAGAAGAACTTTTCACTGG
	TTCTCCTTTACTCATtctgtaataaattgaaatttaggcg
	GTACCGGTAGAAAAAttaggtccagggaagcattt
pYS136	AGGATGCGGAAAGACATCGCGTTTTAGAGCTAGAAATAGC
	GTCTTTCCGCATCCTCaagacatctcgcaataggaggtg
pYS137	CGAAGGATGCGGAAAGACATCGCTAGTG
	TTTCCGCATCCTTCGTCGCCATATATAACG

pYS138	GAACGATTCGTACGTCTGCCAACGTCTCGTCGTCG
	ACGTACGAATCGTTCTAATAGGCGACGAAGGATGCGGAAA
pYS139	GATCAGAACGATTCGGACGTGTTTTAGAGCTAGAAATAGC
	CGAATCGTTCTGATCCaagacatctcgcaataggaggtg
pYS140	CGTCGCCGATCAGAACGATTGTTTTAGAGCTAGAAATAGC
	TTCTGATCGGCGACGCaagacatctcgcaataggaggtg
pYS36	tagcattcgtagaattccaac
	AGAGCCGCTTCCACTACCTTTGTATAGTTCATCCATGCC
	AGTGGAAGCGGCTCTATGAGCGACGACGAGACGTTGG
	ATTCTACGAATGCTACAGATTTTTCAAGACTAGGAAACCAG
pYS379	tatgtgttgtgatgtcac
	tagcattcgtagaattccaac
	acatcacaacacataAATCGAGTGCCATACGTGAAG
	attctacgaatgctaATTGACACGATCTGTTAGTGGCT
pYS38	tagcattcgtagaattccaac
	attctacgaatgctaCTGCTTGAAATACTGTTGTTCG
pYS40	tagcattcgtagaattccaac
	attctacgaatgctaCTCAGAGATCTCCTCTTCGACT
pYS402	acatcacaacacataATTGACACGATCTGTTAGTGGCT
	attctacgaatgctaAATCGAGTGCCATACGTGAAG
	tatgtgttgtgatgtcac
	tagcattcgtagaattccaac
pYS410	agactatgacgtcaaTTTTTCTACCGGTACCCTCAA
	agaaaaatctccaagATGTCGGACACAATTCTTGG
	ttgacgtcatagtcttttagaatggg
	cttggagatttttctggaattaaggtg
pYS436	gtcgacagcgtttcgTGGCGTAATCATGGTCATACTAGT
	ACAGCTTGTCTGTAAGTACCCTCTAGTCAAGGCCT
	cgaaacgctgtcgacttcaac
	TTACAGACAAGCTGTGACCG
pYS526	gtcgacagcgtttcgTGGCGTAATCATGGTCATACTAGT
	ACAGCTTGTCTGTAAGTACCCTCTAGTCAAGGCCT
	cgaaacgctgtcgacttcaac
	TTACAGACAAGCTGTGACCG
pYS527	gtcgacagcgtttcgTGGCGTAATCATGGTCATACTAGT
	ACAGCTTGTCTGTAAGTACCCTCTAGTCAAGGCCT
	cgaaacgctgtcgacttcaac
	TTACAGACAAGCTGTGACCG
pYS56	tagcattcgtagaattccaac
	AGAGCCGCTTCCACTACCTTTGTATAGTTCATCCATGCC
	AGTGGAAGCGGCTCTATGGAATTACACATTTGGCCGT
	attctacgaatgctaCTCAGAGATCTCCTCTTCGACT
pYS57	tagcattcgtagaattccaac

	AGAGCCGCTTCCACTACCTTTGTATAGTTCATCCATGCC
	AGTGGAAGCGGCTCTATGAGCAGCTCCGGGGTGATAA
	attctacgaatgctaCTGCTTGAAATACTGTTGTTCG
pYS59	CTCCGGGGTGATAACCCAATGTTTTAGAGCTAGAAATAGC
	GTTATCACCCCGGAGCaagacatctcgcaataggaggtg
pYS60	CGATGAATGCAGCTCAGGATGTTTTAGAGCTAGAAATAGC
	GAGCTGCATTCATCGCaagacatctcgcaataggaggtg
pYS79	tagcattcgtagaattccaac
	attctacgaatgctaCAGATTTTTCAAGACTAGGAAACC
pYS86	TTGGATGACTCTTACTGGGCGTTTTAGAGCTAGAAATAGC
	GTAAGAGTCATCCAACaagacatctcgcaataggaggtg
pYS87	CGTCCTTGGAGATCGCCGAGGTTTTAGAGCTAGAAATAGC
	CGATCTCCAAGGACGCaagacatctcgcaataggaggtg
pYS88	CTGCATCGATAACCACGTCTGTTTTAGAGCTAGAAATAGC
	TGGTTATCGATGCAGCaagacatctcgcaataggaggtg
pYS163	GAATTCCGGGGATCCCAGGG
	CCGGGTCGACTCGAGCGG
	GGATCCCCGGAATTCGAATTACACATTTGGCCGTCAG
	CTCGAGTCGACCCGGTCACGCATCCCGCATTGGAGGCT
pYS186	GAATTCCGGGGATCCCAGGG
	CCGGGTCGACTCGAGCGG
	GGATCCCCGGAATTCAGCGACGACGAGACGTTGGC
	CTCGAGTCGACCCGGTCAATTCGAGTCGTTGAGGTAG
pYS190	ACGGAGCTCGAATTCGGATCC
	AAGCTTGCGGCCGCACT
	GAATTCGAGCTCCGTAGCGACGACGAGACGTTGGC
	TGCGGCCGCAAGCTTTCAATTCGAGTCGTTGAGGTAG
pYS191	ACGGAGCTCGAATTCGGATCC
	AAGCTTGCGGCCGCACT
	GAATTCGAGCTCCGTAGCAGCTCCGGGGTGATAA
	TGCGGCCGCAAGCTTTTACTGCTTGAAATACTGTTGTTCG
pYS213	GAATTCCGGGGATCCCAGGG
	CCGGGTCGACTCGAGCGG
	GGATCCCCGGAATTCAGCGACGACGAGACGTTGGC
	CTCGAGTCGACCCGGTCAATTCGAGTCGTTGAGGTAG
pYS250	CATATGTATATCTCCTTCTTATACTTAAC
	TCTGGTAAAGAAACCGCTGCT
	GGTTTCTTTACCAGATGCGGCCGCAAGCTT
	GGAGATATACATATGAGCGATAA
pYS251	TGCCATATGTATATCTCCTTCTTATAC
	TCTGGTAAAGAAACCGCTGCT
	GGTTTCTTTACCAGATGCGGCCGCAAGCTT
	GGAGATATACATATGAGCGATAA

pYS254	CATATGTATATCTCCTTCTTATACTTAAC
	TCTGGTAAAGAAACCGCTGCT
	GGAGATATACATATGTCACCTATACTAGGTTATTGGAAA
	GGTTTCTTTACCAGACTCGAGTCGACCCGG
pYS339	ACGGAGCTCGAATTCGGATCC
	AAGCTTGCGGCCGCACT
	GAATTCGAGCTCCGTGATTACCAATTACCGACTCG
	TGCGGCCGCAAGCTTttaAGTGTGATCTGCTCCTCC
pYS372	GAATTCCGGGGATCCCAGGG
	GGATCCCCGGAATTCCCGGGTCGACTCGAGCGG
pYS110	GGTATCGATAAGCTTGATATCGAATTC
	GTCGACCTCGAGGGGGGG
	AAGCTTATCGATACCTACCCATACGATGTTCCTGAC
	CCCCTCGAGGTCGACAGCGTAATCTGGAACGTCAT
pYS111	CCCCTCGAGGTCGACGGT
	GGGCCCGGTACCTTAATTAATTAAG
	GTCGACCTCGAGGGGAGCGACGACGAGACGTTGGC
	TAAGGTACCGGGCCCTTAATTCGAGTCGTTGAGGTAG
pYS112	CCCCTCGAGGTCGACGGT
	GGGCCCGGTACCTTAATTAATTAAG
	GTCGACCTCGAGGGGGAATTACACATTTGGCCGTCAG
	TAAGGTACCGGGCCCTCACGCATCCCGCATTGGAGGCT
pYS113	CCCCTCGAGGTCGACGGT
	GGGCCCGGTACCTTAATTAATTAAG
	GTCGACCTCGAGGGGAGCAGCTCCGGGGTGATAA
	TAAGGTACCGGGCCCTTACTGCTTGAAATACTGTTGTTCG
pYS114	CCCCTCGAGGTCGACGGT
	GGGCCCGGTACCTTAATTAATTAAG
	GTCGACCTCGAGGGGAGCCCAACTCCAACTCGAAAC
	TAAGGTACCGGGCCCTTATCTTCTTGATAAAACACCGTGG
pYS115	GGGCCCGGTACCTTAATTAATTAAGGTA
	CCCCTCGAGGTCGACAGC
	GTCGACCTCGAGGGGAGCGACGACGAGACGTTGGC
	TAAGGTACCGGGCCCTTAATTCGAGTCGTTGAGGTAG
pYS116	GGGCCCGGTACCTTAATTAATTAAGGTA
	CCCCTCGAGGTCGACAGC
	GTCGACCTCGAGGGGGAATTACACATTTGGCCGTCAG
	TAAGGTACCGGGCCCTCACGCATCCCGCATTGGAGGCT
pYS117	GGGCCCGGTACCTTAATTAATTAAGGTA
	CCCCTCGAGGTCGACAGC
	GTCGACCTCGAGGGGAGCAGCTCCGGGGTGATAA
	TAAGGTACCGGGCCCTTACTGCTTGAAATACTGTTGTTCG
pYS175	CCCCTCGAGGTCGACGGT

	GGGCCCGGTACCTTAATTAATTAAG
	GTCGACCTCGAGGGGGAAAATCTCATTCCTGTCGTC
	TAAGGTACCGGGCCCTCACCAAACTTGTGTTTCTCTCA
