# Supplementary material for: Metaxins are core components of mitochondrial transport adaptor complexes
Source: Nat Commun. 2021 Jan 4;12:83. doi: 10.1038/s41467-020-20346-2 (PMC7782850; doi:10.1038/s41467-020-20346-2)
Supplement: Supplementary file 5 — Description of Additional Supplementary Files [file 41467_2020_20346_MOESM5_ESM.docx]

Description of Additional Supplementary information

Title **Extended Data movie 1**

Description: Time-lapse imaging of mitochondria dynamic in PVD neuron of wild type. Mitochondria are visualized by TOMM-20::GFP. The anterior of the animal is toward the left. Frames were taken with 6 seconds interval for 30 min. The display rate is 7 frames per second. The time stamp on the movie indicates minutes:seconds. scale bar: 10 μm.

Title **Extended Data movie 2**

Description**:** Time-lapse imaging of mitochondria dynamic in PVD neuron of *mtx-2* mutant. Mitochondria are visualized by TOMM-20::GFP. The anterior of the animal is toward the left. Frames were taken with 6 seconds interval for 30 min. The display rate is 7 frames per second. The time stamp on the movie indicates minutes:seconds. scale bar: 10 μm.
