# Supplementary material for: Metaxins are core components of mitochondrial transport adaptor complexes
Source: Nat Commun. 2021 Jan 4;12:83. doi: 10.1038/s41467-020-20346-2 (PMC7782850; doi:10.1038/s41467-020-20346-2)
Supplement: Supplementary file 6 — Reporting Summary [file 41467_2020_20346_MOESM6_ESM.pdf]

nature research

Corresponding author(s): Wei Feng; Xiangming Wang; Kang Shen

Last updated by author(s): Nov 10, 2020

Reporting Summary

Nature Research wishes to improve the reproducibility of the work that we publish. This form provides structure for consistency and transparency in reporting. For further information on Nature Research policies, see Authors & Referees and the Editorial Policy Checklist.

Statistics

For all statistical analyses, confirm that the following items are present in the figure legend, table legend, main text, or Methods section.

n/a	Confirmed	
☐	☒	The exact sample size (*n*) for each experimental group/condition, given as a discrete number and unit of measurement
☐	☒	A statement on whether measurements were taken from distinct samples or whether the same sample was measured repeatedly
☐	☒	The statistical test(s) used AND whether they are one- or two-sided<br>*Only common tests should be described solely by name; describe more complex techniques in the Methods section.*
☒	☐	A description of all covariates tested
☐	☒	A description of any assumptions or corrections, such as tests of normality and adjustment for multiple comparisons
☐	☒	A full description of the statistical parameters including central tendency (e.g. means) or other basic estimates (e.g. regression coefficient) AND variation (e.g. standard deviation) or associated estimates of uncertainty (e.g. confidence intervals)
☐	☒	For null hypothesis testing, the test statistic (e.g. *F*, *t*, *r*) with confidence intervals, effect sizes, degrees of freedom and *P* value noted<br>*Give P values as exact values whenever suitable.*
☒	☐	For Bayesian analysis, information on the choice of priors and Markov chain Monte Carlo settings
☒	☐	For hierarchical and complex designs, identification of the appropriate level for tests and full reporting of outcomes
☒	☐	Estimates of effect sizes (e.g. Cohen's *d*, Pearson's *r*), indicating how they were calculated

Our web collection on statistics for biologists contains articles on many of the points above.

Software and code

Policy information about availability of computer code

Data collection	All confocal images were acquired using Zeiss Axio Observer Z1 microscope (equipped with an Andor camera and a spinning-disk confocal scan head), and processed by Image J 1.48g4.<br>Mitochondrial distribution quantification was performed using an Axio Imager M2 microscope (Carl Zeiss).
Data analysis	GraphPad Prism (version: 9.0.0.121) was used for analyzing and graphing.

For manuscripts utilizing custom algorithms or software that are central to the research but not yet described in published literature, software must be made available to editors/reviewers. We strongly encourage code deposition in a community repository (e.g. GitHub). See the Nature Research guidelines for submitting code & software for further information.

Data

Policy information about availability of data

All manuscripts must include a data availability statement. This statement should provide the following information, where applicable:

- Accession codes, unique identifiers, or web links for publicly available datasets
- A list of figures that have associated raw data
- A description of any restrictions on data availability

The quantification analyses in all figures have associated raw data. The raw data are available from the corresponding author upon reasonable request.

Field-specific reporting

Please select the one below that is the best fit for your research. If you are not sure, read the appropriate sections before making your selection.

☒ Life sciences ☐ Behavioural & social sciences ☐ Ecological, evolutionary & environmental sciences

For a reference copy of the document with all sections, see nature.com/documents/nr-reporting-summary-flat.pdf

Life sciences study design

All studies must disclose on these points even when the disclosure is negative.

Sample size	The sample size and the statistical method are indicated in the figure legends or EXPERIMENTAL PROCEDURES section.<br>For mitochondria distribution statistic, sample size is determined according to the published literature. PMID: 24631238 and 28009276.<br>For the mitochondrial dynamic quantification, sample size is determined according to the published literature. PMID: 24995978 and 19135897.<br>For the quantification of PVD dendrite number, sample size is determined according to the published literature. PMID: 29738713 and 22138642.
Data exclusions	No data were excluded from the analysis.
Replication	All statistical experiments were performed in at least two independent experiments with similar results. All attempts at replication were successful.
Randomization	For all the worms (WT or mutants), they are homogenous with same genotypes. No big difference was observed between individual worm. We chose worms randomly to do the observation and quantification.
Blinding	The investigators were blinded to group allocations.

Reporting for specific materials, systems and methods

We require information from authors about some types of materials, experimental systems and methods used in many studies. Here, indicate whether each material, system or method listed is relevant to your study. If you are not sure if a list item applies to your research, read the appropriate section before selecting a response.

Materials & experimental systems

n/a	Involved in the study	
☐	☒	Antibodies
☐	☒	Eukaryotic cell lines
☒	☐	Palaeontology
☐	☒	Animals and other organisms
☒	☐	Human research participants
☒	☐	Clinical data

Methods

n/a	Involved in the study	
☒	☐	ChIP-seq
☒	☐	Flow cytometry
☒	☐	MRI-based neuroimaging

Antibodies

Antibodies used

1. Anti-HA antibody produced in rabbit, affinity isolated antibody from Sigma-Aldrich, Catalog Number H6908.
2. Anti-FLAG antibody produced in rabbit, affinity isolated antibody from Sigma-Aldrich, Product Number F7425.
3. mouse anti-Metaxin-1 (sc-514469, Santa Cruz Biotechnology).
4. mouse anti-Metaxin-2 (SC-514231, Santa Cruz Biotechnology).
5. rabbit anti-Trak1 (HPA005853, Sigma-Aldrich).
6. rabbit anti-Trak2 (PA5-31459, Thermo Fisher).
7. mouse anti-ATP5β (AB14730, AbCam).
8. mouse anti-β-actin (A00702, Genscript).
9. mouse anti-Tom20 (sc-17764, Santa Cruz Biotechnology).
10. mouse anti-Miro1 (ab188029, AbCam).
11. mouse anti-Miro1 (WH0055288M1, Sigma-Aldrich).
12. rabbit anti-GFP (50430-2-AP, Proteintech).
13. rabbit anti-β-actin (ab52614, AbCam).

Validation

1. For anti-HA antibody (Sigma-Aldrich, H6908), detailed information can be found online, https://www.sigmaaldrich.com/catalog/product/sigma/h6908. As showed in the antibody description from Sigma, this HA antibody has good performance for detection of HA-tagged fusion protein from transfected cells by western blot (Figure 1, adapted from sigma-aldrich website)

Figure 1. https://www.sigmaaldrich.com/catalog/product/sigma/h6908.
Immunoblotting
Whole extract of human HEK-293T cells overexpressing N-terminal HA tagged fusion protein was separated on SDS-PAGE and probed with Rabbit Anti-HA (Cat. No. H6908). The antibody was developed using Goat Anti-Rabbit IgG-Peroxidase and a chemiluminescent substrate.
Lanes
1. 0.5 μg/mL antibody
2. 1 μg/mL antibody
3. negative control: no first Antibody

2. For anti-FLAG antibody (Sigma-Aldrich, F7425), detailed information can be found online, https://www.sigmaaldrich.com/catalog/product/sigma/f7425; As showed in the antibody description from Sigma, this FLAG antibody has good performance for detection of FLAG-tagged fusion protein from transfected cells by western blot (Figure 2, adapted from sigma-aldrich website)

Figure 2. https://www.sigmaaldrich.com/catalog/product/sigma/f7425
Immunoblotting
Whole extract of human HEK-293T cells over-expressing N-Terminal Flag tagged fusion protein was separated on SDS-PAGE and probed with Rabbit Anti-FLAG® (Cat. No. F7425). The antibody was developed using Goat Anti-Rabbit IgG-Peroxidase and a chemiluminescent substrate.
Lanes
1. 0.4 μg/mL antibody
2. Negative Control: without first antibody
For 3-6 antibodis were validated using RNAi knockdown in Figure S6. Anti-ATP5β and anti-β-actin are standard antibodies and were validated by the respective vendors.

Eukaryotic cell lines

Policy information about cell lines

Cell line source(s)	HEK293T from ATCC<br>iPSC line (healthy male aged 62)
Authentication	HEK293T cell line was authenticated by ATCC. iPSC line was authenticated in Hsieh et al., 2016. PMID: 27618216.
Mycoplasma contamination	We have confirmed in our lab, and the result showed no mycoplasma contamination.
Commonly misidentified lines<br>(See ICLAC register)	No commonly misidentified cell lines were used in the study.

Animals and other organisms

Policy information about studies involving animals; ARRIVE guidelines recommended for reporting animal research

Laboratory animals

We used hermaphrodite of wild type or mutant worms, which are all from the wild type strain Bristol N2.
For mitochondrial distribution quantification, worms at day 1 stage were examined.
for mitochondrial dynamic experiments, L3 or early L4 stage worms were used.
for PVD dendrite degeneration phenotype, Day 1, 2, 4 and 6 worms were used.
drp-1(tm1108)dhc-1(or195ts)unc-116(e2310)klc-2(km11)klc-1(ok2609)wyIs592wyIs594miro-1(wy50180)trak-1(wy50182);wyIs592miro-1(wy50180);wyIs592dli-1(wy50053)dli-1(wy50053);wyIs50054drp-1(tm1108);wyIs50054klc-2(km11);wyIs50054miro-1(wy50180);wyIs50054trak-1(wy50182);wyIs50054unc-116(e2310);wyIs50054trak-1(wy50182);miro-1(wy50180);wyIs50054trak-1(wy50182);dli-1(wy50235);wyIs50054 unc-116(e2310);miro-1(wy50180);wyIs50054,trak-1(wy50182);unc-116(e2310);wyIs50054miro-1(wy50285 [gfp::miro-1])miro-1(wy50260)miro-1(wy50233)
trak-1(wy50182)mtx-1(wy50272)mtx-1(wy50286)mtx-2(wy50250);wyIs50054mtx-2(wy50266)mtx-2(wy50250)mtx-2(wy50256)mtx-2(wy50266);wyIs50054mtx-2(gk444);wyIs50054miro-1(wy50260);wyIs50054mtx-1(wy50286);wyIs50054miro-1(wy50233);wyIs50054dli-1(wy50235)mtx-1(wy50272);wyIs50054wySi50001;unc-119(ed4)wySi50002;unc-119(ed4)wySi50004;unc-119(ed4), mtx-1(ok3155);wyIs50054mtx-2(wy50266);wyIs594 wyIs50054wyIs50091
miro-1(wy50180);wyIs50054;wySi50004;unc-119(ed4)trak-1(wy50182);wyIs50054;wySi50004;unc-119(ed4)trak-1(wy50182);
mtx-2(wy50266);wyIs50054trak-1(wy50182);mtx-1(wy50272);wyIs50054mtx-1(wy50272);miro-1(wy50180);wyIs50054mtx-2(wy50266);miro-1(wy50180);wyIs50054mtx-2(wy50266);mtx-1(wy50272);wyIs50054mtx-2(wy50266);wyIs50054;wySi50004;unc-119(ed4)dli-1(wy50235);wyIs50054 klc-1(ok2609);wyIs50054 dhc-1(or195ts);wyIs50054trak-1(wy50182);wySi50001;unc-119(ed4) trak-1(wy50182);wySi50002;unc-119(ed4)miro-1(wy50180);wySi50001;unc-119(ed4)mtx-1(wy50266);wySi50002;
unc-119(ed4)wyEx50750mtx-1(50272);wySi50001;unc-119(ed4), mtx-1(wy50272);wyIs50091
mtx-1(wy50272);miro-1(wy50285) [gfp::miro-1]), mtx-2(wy50266);wySi50001;unc-119(ed4) mtx-2(wy50266);wyIs50091
mtx-2(wy50266);miro-1(wy50285[gfp::miro-1])mtx-2(wy50266);miro-1(wy50180);wyIs50091mtx-1(wy50272);wyIs592mtx-2(wy50266);wySi50002;unc-119(ed4)klc-2(km11);wyIs50091miro-1(wy50180);wyIs50091trak-1(wy50182);wyIs50091,
unc-116(e2310);wyIs50091trak-1(wy50182);miro-1(wy50180);wyIs50091mtx-1(wy50272);miro-1(wy50180);wyIs50091trak-1(wy50182);mtx-1(wy50272);wyIs50091trak-1(wy50182);mtx-2(wy50266);wyIs50091mtx-2(wy50266);miro-1(wy50180);mtx-1(wy50272);wyIs50091mtx-1(wy50272);wySi50002;unc-119(ed4)miro-1wy50180);wySi50002;unc-119(ed4)mtx-2(wy50266);
mtx-1(wy50272);wyIs50091mtx-2(wy50266);mtx-1(wy50272);miro-1(wy50180);wyIs50054dli-1(wy50235);wyIs50091klc-1(ok2609);wyIs50091wyEx50521wyEx50801wyIs50082wyIs50082;wySi50001;unc-119(ed4)wyIs50082;wySi50002;unc-119(ed4)wyEx50801;miro-1(wy50285[gfp::miro-1])wyIs50082;wySi50002;mtx-2(wy50266);unc-119(ed4)
wyEx50802mtx-2(wy50256);
wyIs50054wySi50085;unc-119(ed4)wySi50086;unc-119(ed4)wySi50085;unc-119(ed4);miro-1(wy50180)wySi50086;unc-119(

ed4);miro-1(wy50180)wySi50085;unc-119(ed4);mtx-2(wy50266)wySi50086;unc-119(ed4);mtx-2(wy50266)gop-3(tm3269)/hT2 [qIs48];wyIs50054gop-3(tm3269)/hT2 [qIs48];wySi50001;unc-119(ed4)wyIs50082;gop-3(tm3269)/hT2 [qIs48];wySi50001;unc-119(ed4)mtx-2(gk444)mtx-1(ok3155)

Wild animals	We do not use wild animals in our study.
Field-collected samples	We do not use Field-collected samples in our study.
Ethics oversight	No ethical approval was required because we only used Caenorhabditis elegans and cultured cells.

Note that full information on the approval of the study protocol must also be provided in the manuscript.
